# Supplementary material for: The changing epidemiology of human monkeypox—A potential threat? A systematic review
Source: PLoS Negl Trop Dis. 2022 Feb 11;16(2):e0010141. doi: 10.1371/journal.pntd.0010141 (PMC8870502; doi:10.1371/journal.pntd.0010141)
Supplement: S1 Table — (DOCX) [file pntd.0010141.s001.docx]

**S1 Table. Number of Monkeypox Cases by Decade by Country***

|  | **1970-1979** | **1980-1989** | **1990-1999** | **2000-2009** | **2010-2019** |
| --- | --- | --- | --- | --- | --- |
|  | **Number of Cases** | | | | |
| Africa |  |  |  |  |  |
| DRC | 38 | 343 | 511 | 10,027 | 18,788 |
| Nigeria | 3 | — | — | — | 181 |
| Liberia | 4 | — | — | — | 6 |
| Cameroon | 1 | 1 | — | — | 3 |
| Côte d’Ivoire | 1 | 1 | — | — | — |
| Sierra Leone | 1 | — | — | — | 2 |
| Gabon | — | 4 | 9 | — | — |
| Central African Republic | — | 8 | — | — | 61 |
| Congo | — | — | — | 73 | 24 |
| South Sudan | — | — | — | 19 | — |
| Other Continents |  |  |  |  |  |
| United States | — | — | — | 47 | — |
| United Kingdom | — | — | — | — | 4 |
| Israel | — | — | — | — | 1 |
| Singapore | — | — | — | — | 1 |

* All data reflect the number of confirmed, probable, and/or possible number of monkeypox cases, except for the Democratic Republic of the Congo (DRC) for the years 2000-2009 and 2010-2019, since as of the year 2000, the number of suspected cases was primarily reported by the DRC.
