# Supplementary material for: The changing epidemiology of human monkeypox—A potential threat? A systematic review
Source: PLoS Negl Trop Dis. 2022 Feb 11;16(2):e0010141. doi: 10.1371/journal.pntd.0010141 (PMC8870502; doi:10.1371/journal.pntd.0010141)
Supplement: S2 Table — (DOCX) [file pntd.0010141.s002.docx]

#### Table S2. Number of suspected cases versus confirmed, probable, and/or possible cases

| Author, year (citation) | Study period | Suspected cases (N) | Confirmed, probable, and/or possible cases (n) | Confirmed, probable, and/or possible cases (%) |
| --- | --- | --- | --- | --- |
| *Cameroon* | | | | |
| *WHO, 2018 (67)* | *April - June 2018* | *36 (nr of tested NR)* | *1* | *2.8* |
| *WHO, 2020 (65)* | *Sep 2019* | *1 (nr of tested NR)* | *0* | *0* |
| *Central African Republic* | | | | |
| Kalthan, 2018 (33) | Aug - Oct 2016 | 23 of which 7 were tested | 3 | 42.9 |
| *WHO, 2016 (62)* | *Sep - Oct 2016* | *27 (nr of tested NR)* | *3* | *11.1* |
| *WHO, 2017 (60)* | *Feb - April 2017* | *47 (nr of tested NR)* | *5* | *10.6* |
| *WHO, 2017 (61)* | *April-June 2017* | *3 (nr of tested NR)* | *2* | *66.7* |
| *WHO, 2019 (59)* | *March 2018 - June 2019* | *38 (nr of tested NR)* | *25* | *65.8* |
| *Democratic Republic of the Congo* | | | | |
| Meyer, 2002 (24) | Feb - Aug 2001 | 31 of which 14 were tested | 7 | 50 |
| Rimoin, 2007 (27) | Jan 2001 - Dec 2004 | 2734 of which 136 were tested | 51 | 37.5 |
| Hoff, 2017 (19) | Nov 2005 - Jan 2008 | 1158 (all were tested) | 785 | 67.8 |
| Nolen, 2016 (25) | July - Dec 2013 | 63 (unclear how many were tested) | 39 | 61.9 |
| McCollum, 2015 (23) | 2011-2014 | 6 (all were tested) | 3 | 50.0 |
| *Laudisoit, 2016 (63)* | *Jan - March 2016* | *160 of which 12 were tested* | *7* | *58.3* |
| *WHO, 2020 (66)* | *Jan - Sep 2020* | *4594 (nr of tested NR)* | *39* | *0.9* |
| *Liberia* | | | | |
| *WHO, 2018 (73)* | *Nov 2016 - Dec 2017* | *16 (nr of tested NR)* | *2* | *12.5* |
| *Nigeria* | | | | |
| Yinka-Ogunleye, 2019 (44) | Sep 2017 – Sep 2018 | 276 (of which 253 were tested) | 122 | 44.2 |
| *Republic of the Congo* | | | | |
| Learned, 2005 (46) | April - June 2003 | 12 (all were tested) | 11 | 91.7 |
| Reynolds, 2013 (47) | April - Nov 2010 | 10 of which 4 were tested | 2 | 50.0 |
| Doshi, 2019 (15) | Jan - 5 April 2017 | 43 (unclear how many were tested) | 22 | 51.2 |
| *WHO, 2017 (71)* | *Jan - Sep 2017* | *88 (nr of tested not reported)* | *8* | *9.1* |
| *WHO, 2019 (70)* | *March 2019* | *9 (nr of tested NR)* | *2* | *22.2* |
| *South Sudan* | | | | |
| Formenty, 2010 (58) | Sep – Dec 2005 | 49 of which 31 were tested | 19 | 61.3 |
| *United States* | | | | |
| CDC, 2003 *(6)* (36-39) | June – July 2003 | 96 (likely all were tested) | 47 | 49.0 |

#### Note: Citation numbers reflect those that are in the main manuscript text, and those in italics refer to grey literature sources. CDC = United States Centers for Disease Control and Prevention; nr of tested NR = number of suspected individuals tested is not reported.

**References** (listed in alphabetical order; citation numbers in the Table reflect those that are in the main manuscript text for ease of identification)

Centers for Disease Control and Prevention. Monkeypox. Available from: <https://www.cdc.gov/poxvirus/monkeypox/index.html>

Centers for Disease Control and Prevention. Update: multistate outbreak of monkeypox--Illinois, Indiana, Kansas, Missouri, Ohio, and Wisconsin, 2003. MMWR Morb Mortal Wkly Rep. 2003;52(24):561-564.

Centers for Disease Control and Prevention. Update: multistate outbreak of monkeypox--Illinois, Indiana, Kansas, Missouri, Ohio, and Wisconsin, 2003. MMWR Morb Mortal Wkly Rep. 2003;52(25):589-590.

Centers for Disease Control and Prevention. Update: multistate outbreak of monkeypox--Illinois, Indiana, Kansas, Missouri, Ohio, and Wisconsin, 2003. MMWR Morb Mortal Wkly Rep. 2003;52(26):616-618.

Centers for Disease Control and Prevention. Update: multistate outbreak of monkeypox--Illinois, Indiana, Kansas, Missouri, Ohio, and Wisconsin, 2003. MMWR Morb Mortal Wkly Rep. 2003;52(27):642-646.

Doshi RH, Guagliardo SAJ, Doty JB, Babeaux AD, Matheny A, Burgado J, et al. Epidemiologic and ecologic investigations of monkeypox, Likouala Department, Republic of the Congo, 2017. Emerg Infect Dis. 2019;25(2):281-289.

Formenty P, Muntasir MO, Damon I, Chowdhary V, Opoka ML, Monimart C, et al. Human monkeypox outbreak caused by novel virus belonging to Congo Basin clade, Sudan, 2005. Emerg Infect Dis. 2010;16(10):1539-1545.

Hoff NA, Morier DS, Kisalu NK, Johnston SC, Doshi RH, Hensley LE, et al. Varicella coinfection in patients with active monkeypox in the Democratic Republic of the Congo. Ecohealth. 2017;14(3):564-574.

Kalthan E, Tenguere J, Ndjapou SG, Koyazengbe TA, Mbomba J, Marada RM, et al. Investigation of an outbreak of monkeypox in an area occupied by armed groups, Central African Republic. Med Mal Infect. 2018;48(4):263-268.

Laudisoit A, Komba M, Akonda I. Scientific report research bushmeat and monkeypox Yahuma health zone–Aketi health zone-Bombongolo health area. DRC. 2016. Available from: https://www.researchgate.net/profile/Anne-Laudisoit/publication/312332722_Monkeypox_Outbreak_Investigation_Aketi_2016/links/587b4d0108aed3826ae838d0/Monkeypox-Outbreak-Investigation-Aketi-2016.pdf

Learned LA, Reynolds MG, Wassa DW, Li Y, Olson VA, Karem K, et al. Extended interhuman transmission of monkeypox in a hospital community in the Republic of the Congo, 2003. Am J Trop Med Hyg. 2005;73(2):428-434.

McCollum AM, Nakazawa Y, Ndongala GM, Pukuta E, Karhemere S, Lushima RS, et al. Case report: Human monkeypox in the Kivus, a conflict region of the Democratic Republic of the Congo. Am J Trop Med Hyg. 2015;93(4):718-721.

Meyer H, Perrichot M, Stemmler M, Emmerich P, Schmitz H, Varaine F, et al. Outbreaks of disease suspected of being due to human monkeypox virus infection in the Democratic Republic of Congo in 2001. J Clin Microbiol. 2002;40(8):2919-2921.

Nolen LD, Osadebe L, Katomba J, Likofata J, Mukadi D, Monroe B, et al. Extended human-to-human transmission during a monkeypox outbreak in the Democratic Republic of the Congo. Emerg Infect Dis. 2016;22(6):1014-1021.

Reynolds MG, Emerson GL, Pukuta E, Karhemere S, Muyembe JJ, Bikindou A, et al. Detection of human monkeypox in the Republic of the Congo following intensive community education. Am J Trop Med Hyg. 2013;88(5):982-985.

Rimoin AW, Kisalu N, Kebela-Ilunga B, Mukaba T, Wright LL, Formenty P, et al. Endemic human monkeypox, Democratic Republic of Congo, 2001-2004. Emerg Infect Dis. 2007;13(6):934-937.

World Health Organization. Monkeypox in Central African Republic [press release]. Reliefweb, October 14, 2016. Available from: <https://reliefweb.int/report/central-african-republic/monkeypox-central-african-republic>

World Health Organization. Regional Office for Africa, Health Emergencies Programme. 2017. Weekly Bulletin on Outbreaks and other Emergencies: Week 21: 20 – 26 May 2017. Available from: https://apps.who.int/iris/handle/10665/255579

World Health Organization. Regional Office for Africa, Health Emergencies Programme. 2017. Weekly Bulletin on Outbreak and other Emergencies: Week 31: 29 July – 04 August 2017. Available from: https://apps.who.int/iris/handle/10665/258688

World Health Organization. Regional Office for Africa, Health Emergencies Programme. 2017. Weekly Bulletin on Outbreaks and other Emergencies: Week 41: 7 – 13 October 2017. Available from: https://apps.who.int/iris/handle/10665/259263

World Health Organization. Regional Office for Africa, Health Emergencies Programme. 2018. Weekly Bulletin on Outbreaks and other Emergencies: Week 1: 30 December 2017-5 January 2018. Available from: https://apps.who.int/iris/handle/10665/259809

World Health Organization. Regional Office for Africa. 2018. Weekly Bulletin on Outbreak and other Emergencies: Week 31: 28 July - 3 August 2018. Available from: https://apps.who.int/iris/handle/10665/273631

World Health Organization. Regional Office for Africa. 2019. Weekly Bulletin on Outbreak and other Emergencies: Week 21: 20 - 26 May 2019. Available from: https://apps.who.int/iris/handle/10665/324950

World Health Organization. Regional Office for Africa. 2019. Weekly Bulletin on Outbreak and other Emergencies: Week 31: 29 July - 04 August 2019. Available from: https://apps.who.int/iris/handle/10665/326159

World Health Organization. Regional Office for Africa. 2020. Weekly Bulletin on Outbreak and other Emergencies: Week 01: 30 December 2019 - 05 January 2020. Available from: https://apps.who.int/iris/handle/10665/330353

World Health Organization. Regional Office for Africa. 2020. Weekly Bulletin on Outbreak and other Emergencies: Week 41: 05 - 11 October 2020. Available from: https://apps.who.int/iris/handle/10665/336026

Yinka-Ogunleye A, Aruna O, Dalhat M, Ogoina D, McCollum A, Disu Y, et al. Outbreak of human monkeypox in Nigeria in 2017-18: a clinical and epidemiological report. Lancet Infect Dis. 2019;19(8):872-879.
