# Supplementary material for: The changing epidemiology of human monkeypox—A potential threat? A systematic review
Source: PLoS Negl Trop Dis. 2022 Feb 11;16(2):e0010141. doi: 10.1371/journal.pntd.0010141 (PMC8870502; doi:10.1371/journal.pntd.0010141)
Supplement: S3 Table — (DOCX) [file pntd.0010141.s003.docx]

#### Table S3 Secondary attack rate

| Author, year (citation) | Secondary attack rate % (n/N) | Comments |
| --- | --- | --- |
| *Cameroon* | | |
| Tchokoteu, 1991 (50) | 0 | Denominator of household members was 20, number of neighbors unknown |
| *Central African Republic* | | |
| Besombes, 2019 (30) | 0 (0/33) |  |
| *Democratic Republic of the Congo* | | |
| Breman, 1980 (5) | 7.5 (3/40)  3.3 (4/123) | 7.5% refers to very close family members; 3.3% refers to all susceptible contacts. Note that all susceptible contacts possibly include contacts from 9 cases from other countries; data for secondary attack rate not presented per country. |
| Jezek, 1988 (22) | 3 (69/2278) | First generation contacts |
| Jezek, 1986 (20) | 10.2 (4/39) |  |
| Aplogan, 1997 (14) | 8 | Editorial note: secondary attack rates were estimated to be 8% (95% CI 5-12%), no further details provided |
| Nolen, 2016 (25) | 50 | Median attack rate in the 16 households: 50% (range 50-100) |
| McCollum, 2015 (23) | 0 (0/30) | Contacts of one case |
| *Gabon* | | |
| Meyer, 1991 (54) | 0.3 (1/292) |  |
| *Israel* | | |
| Erez, 2019 (57) | 0 (0/16) |  |
| *Ivory Coast (C*ô*te d’Ivoire)* | | |
| Breman, 1977 (51) | 0 |  |
| Merouze, 1983 (52) | 0 (0/7) |  |
| *Liberia* | | |
| Foster, 1972 (42) | 0 (0/44) |  |
|  | 0 (0/23) |  |
|  | 0 (0/23) |  |
|  | 0 (0/136) |  |
| *Sierra Leone* | | |
| Foster, 1972 (42) | 0 (0/30) |  |
| Ye, 2019 (49) | 0 (0/16) |  |
| *Singapore* | | |
| Yong, 2020 (8) | 0 (0/21) |  |
| *United Kingdom* | | |
| Vaughan, 2020 (56) | 0.3 (1/288) |  |

#### Note: Citation numbers reflect those that are in the main manuscript text.

**References** (listed in alphabetical order; citation numbers in the Table reflect those that are in the main manuscript text for ease of identification)

Aplogan A, Mangindula V, Muamba PT, Mwema GN, Okito L, Pebody RG, et al. Human monkeypox -- Kasai Oriental, Democratic Republic of Congo, February 1996-October 1997. MMWR Morb Mortal Wkly Rep. 1997;46(49):1168-1171.

Besombes C, Gonofio E, Konamna X, Selekon B, Grant R, Gessain A, et al. Intrafamily transmission of monkeypox virus, Central African Republic, 2018. Emerg Infect Dis. 2019;25(8):1602-1604.

Breman JG, Nakano JH, Coffi E, Godfrey H, Gautun JC. Human poxvirus disease after smallpox eradication. Am J Trop Med Hyg. 1977;26(2):273-281.

Breman JG, Kalisa R, Steniowski MV, Zanotto E, Gromyko AI, Arita I. Human monkeypox, 1970-79. Bull World Health Organ. 1980;58(2):165-182.

Erez N, Achdout H, Milrot E, Schwartz Y, Wiener-Well Y, Paran N, et al. Diagnosis of imported monkeypox, Israel, 2018. Emerg Infect Dis. 2019;25(5):980-983.

Foster SO, Brink EW, Hutchins DL, Pifer JM, Lourie B, Moser CR, et al. Human monkeypox. Bull World Health Organ. 1972;46(5):569-576.

Jezek Z, Arita I, Mutombo M, Dunn C, Nakano JH, Szczeniowski M. Four generations of probable person-to-person transmission of human monkeypox. Am J Epidemiol. 1986;123(6):1004-1012.

Jezek Z, Grab B, Szczeniowski MV, Paluku KM, Mutombo M. Human monkeypox: secondary attack rates. Bull World Health Organ. 1988;66(4):465-470.

McCollum AM, Nakazawa Y, Ndongala GM, Pukuta E, Karhemere S, Lushima RS, et al. Case report: Human monkeypox in the Kivus, a conflict region of the Democratic Republic of the Congo. Am J Trop Med Hyg. 2015;93(4):718-721.

Merouze F, Lesoin JJ. [Monkeypox: second human case observed in Ivory Coast (rural health sector of Daloa]. Med Trop (Mars). 1983;43(2):145-147.

Meyer A, Esposito JJ, Gras F, Kolakowski T, Fatras M, Muller G. [First appearance of monkey pox in human beings in Gabon]. Med Trop (Mars). 1991;51(1):53-57.

Nolen LD, Osadebe L, Katomba J, Likofata J, Mukadi D, Monroe B, et al. Extended human-to-human transmission during a monkeypox outbreak in the Democratic Republic of the Congo. Emerg Infect Dis. 2016;22(6):1014-1021.

Tchokoteu PF, Kago I, Tetanye E, Ndoumbe P, Pignon D, Mbede J. [Variola or a severe case of varicella? A case of human variola due to monkeypox virus in a child from the Cameroon]. Ann Soc Belg Med Trop. 1991;71(2):123-128.

Vaughan A, Aarons E, Astbury J, Brooks T, Chand M, Flegg P, et al. Human-to-human transmission of monkeypox virus, United Kingdom, October 2018. Emerg Infect Dis. 2020;26(4):782-785.

Ye F, Song J, Zhao L, Zhang Y, Xia L, Zhu L, et al. Molecular evidence of human monkeypox virus infection, Sierra Leone. Emerg Infect Dis. 2019;25(6):1220-1222.

Yong SEF, Ng OT, Ho ZJM, Mak TM, Marimuthu K, Vasoo S, et al. Imported monkeypox, Singapore. Emerg Infect Dis. 2020;26(8):1826-1830.
