# Supplementary material for: The changing epidemiology of human monkeypox—A potential threat? A systematic review
Source: PLoS Negl Trop Dis. 2022 Feb 11;16(2):e0010141. doi: 10.1371/journal.pntd.0010141 (PMC8870502; doi:10.1371/journal.pntd.0010141)
Supplement: S4 Table — (DOCX) [file pntd.0010141.s004.docx]

#### Table S4. Age and sex of confirmed, probable, and/or possible and hospitalised cases from Africa

| Author, year (citation) | Study period | Confirmed cases (n) | Probable cases (n) | Confirmed or probable case (n) | Possible cases (n) | Age median; IQR or range (yrs) | Age case report (yrs) | Age other | Males  n (%) |
| --- | --- | --- | --- | --- | --- | --- | --- | --- | --- |
| ***Cameroon*** | | | | | | | | | |
| Breman, 1980 (5) | 1979 | 1 |  |  |  |  | 3 |  | 0 |
| Tchokoteu, 1991 (50) | Dec 1989 | 1 |  |  |  |  | 7 |  | NR |
| *WHO, 2020 (68)* | *Dec 2019* | *1* | *1* |  |  |  | *1 and NR (mother)* |  | *NR* |
| ***Central African Republic*** | | | | | | | | | |
| Herve, 1989 (31) | Likely 1980s | 2 |  |  |  |  | 8 and 6 |  | 1 (50) |
| Khodakevich, 1985 (34) | Jan 1984 | 6 |  |  |  |  |  | 5 children; 1 of 22 yrs | 22 year- old is a women. Other NR |
| Berthet, 2011 (29) | June 2010 | 2 |  |  |  |  | 14 and 15 |  | 2 (100) |
| Nakouné, 2017 (35); Kalthan 2016 (32) | Dec 2015 - Jan 2016 |  |  | 13 |  | 1-41 |  |  | 7 (53.8) |
| Besombes  2019 (30) | Sep - Oct 2018 | 6 |  |  |  | 4 months – 33 yrs |  |  | 0 |
| ***Democratic Republic of the Congo*** | | | | | | | | | |
| Breman, 1980 (5) | 1970-1979 |  |  | 38 |  | 7 months – 41 yrs |  |  | 19 (50) |
| Jezek, 1988 (21) | 1981-1986 | 338 |  |  |  | 4.4 (3 months - 69 yrs) |  |  | 182 (53.8) |
| Jezek, 1986 (20) | May-July 1983 | 5 |  |  |  | 1-7 |  |  | 4 (80) |
| Mwanbal, 1997 (13) | Feb 1996 - Feb 1997 |  |  |  | 92 |  |  | 25 (27.2%) were ≥15 | 51 (55.4) |
| Aplogan, 1997 (14) | Feb 1996 - Oct 1997 |  | 304 |  | 115 |  |  | 85% were <16 | NR |
| Meyer, 2002 (24) | Feb - Aug 2001 | 7 |  |  |  | 1-30 |  |  | 6 (86) |
| Rimoin, 2007 (27) | Jan 2001 - Dec 2004 | 51 |  |  |  | most were <14 |  |  | 28 (54.9) |
| Rimoin, 2010 (28) | Nov 2005 - Nov 2007 | 760 |  |  |  | 10 (5 days-70 yrs) |  |  | 472 (62.1) |
| Hoff, 2017 (19) | Nov 2005 - Jan 2008 | 785 |  |  |  |  |  | 24.2% were <5 | 480 (61.1) |
| Nolen, 2016 (25) | July - Dec 2013 | 20 | 19 |  |  | 10 (4 months - 68 yrs) |  |  | 36 (57.1) |
| McCollum, 2015 (23) | 2011-2014 | 3 |  |  |  |  | 23-28 |  | 2 (66.7) |
| Eltvedt, 2020 (16) | Dec 2016 |  | 1 |  |  |  | 4 |  | 1 (100) |
| ***Gabon*** | | | | | | | | | |
| Meyer, 1991 (54) | June 1987 | 1 | 3 |  |  | 9 months – 9 years |  |  | 1 (25) |
| No authors, 1992 (53) | Jan, May-July 1991 | 5 |  |  |  | 3-11 |  |  | NR |
| ***Ivory Coast (C***ô***te d’Ivoire)*** | | | | | | | | | |
| Breman, 1977 (51) | Oct 1972 |  | 1 |  |  |  | 5 |  | 1 (100) |
| Merouze, 1983 (52) | Jan 1981 | 1 |  |  |  |  | 3 |  | 0 |
| ***Liberia*** | | | | | | | | | |
| Foster, 1972 (42) | Sep 1970 | 1 |  |  |  |  | 4 |  | 0 |
|  | Sep 1970 | 1 |  |  |  |  | 4 |  | 1 (100) |
|  | Sep 1970 | 1 |  |  |  |  | 6 |  | 0 |
|  | Oct 1970 | 1 |  |  |  |  | 9 |  | 1 (100) |
| ***Nigeria*** | | | | | | | | | |
| Foster, 1972 (42) | April 1971 | 1 |  |  |  |  | 4 |  | 0 |
| Breman, 1980 (5) | April 1971 | 1 |  |  |  |  | 24 |  | 0 |
| Breman, 1980 (5) | Nov 1978 | 1 |  |  |  |  | 35 |  | 1 |
| Yinka-Ogunleye, 2019 (44) | Sep 2017 - Sep 2018 | 118 | 4 |  |  | 29; 14 (5 days – 50 yrs) |  |  | 84 (69) |
| ***Republic of the Congo*** | | | | | | | | | |
| Learned, 2005 (46) | April - June 2003 | 3 | 8 |  |  | 8 |  |  | 8 (73) |
| Reynolds, 2013 (47) | April - Nov 2010 | 2 |  |  |  |  | 7 and 16 |  | 0 |
| Doshi, 2019 (15) | Jan - 5 April 2017 | 7 | 13 |  | 2 | 11.5 (1-40) |  |  | 8 (36.4) |
| ***Sierra Leone*** | | | | | | | | | |
| Foster, 1972 (42) | Dec 1970 | 1 |  |  |  |  | 24 |  | 1 (100) |
| Reynolds, 2019 (48) | March 2014 | 1 |  |  |  |  | 11 months |  | 1 (100) |
| Ye, 2019 (49) | March 2017 | 1 |  |  |  |  | 35 |  | 1 (100) |
| ***South Sudan*** | | | | | | | | | |
| Formenty, 2010 (58) | Sep - Dec 2005 | 10 | 9 |  |  | 8 months – 32 yrs |  |  | 9 (47) |

Note: Citation numbers reflect those that are in the main manuscript text, and those in italics refer to grey literature sources. IQR = interquartile range; NR = not reported; yrs = years.

**References** (listed in alphabetical order; citation numbers in the Table reflect those that are in the main manuscript text for ease of identification)

Aplogan A, Mangindula V, Muamba PT, Mwema GN, Okito L, Pebody RG, et al. Human monkeypox -- Kasai Oriental, Democratic Republic of Congo, February 1996-October 1997. MMWR Morb Mortal Wkly Rep. 1997;46(49):1168-1171.

Berthet N, Nakouné E, Whist E, Selekon B, Burguière AM, Manuguerra JC, et al. Maculopapular lesions in the Central African Republic. Lancet. 2011;378(9799):1354.

Besombes C, Gonofio E, Konamna X, Selekon B, Grant R, Gessain A, et al. Intrafamily transmission of monkeypox virus, Central African Republic, 2018. Emerg Infect Dis. 2019;25(8):1602-1604.

Breman JG, Nakano JH, Coffi E, Godfrey H, Gautun JC. Human poxvirus disease after smallpox eradication. Am J Trop Med Hyg. 1977;26(2):273-281.

Breman JG, Kalisa R, Steniowski MV, Zanotto E, Gromyko AI, Arita I. Human monkeypox, 1970-79. Bull World Health Organ. 1980;58(2):165-182.

Doshi RH, Guagliardo SAJ, Doty JB, Babeaux AD, Matheny A, Burgado J, et al. Epidemiologic and ecologic investigations of monkeypox, Likouala Department, Republic of the Congo, 2017. Emerg Infect Dis. 2019;25(2):281-289.

Eltvedt AK, Christiansen M, Poulsen A. A case report of monkeypox in a 4-year-old boy from the DR Congo: challenges of diagnosis and management. Case Rep Pediatr. 2020;2020:8572596.

Formenty P, Muntasir MO, Damon I, Chowdhary V, Opoka ML, Monimart C, et al. Human monkeypox outbreak caused by novel virus belonging to Congo Basin clade, Sudan, 2005. Emerg Infect Dis. 2010;16(10):1539-1545.

Foster SO, Brink EW, Hutchins DL, Pifer JM, Lourie B, Moser CR, et al. Human monkeypox. Bull World Health Organ. 1972;46(5):569-576.

Herve VMA, Belec L, Yayah G, Georges AJ. Monkeypox in Central Africa. About two strains isolated Central African Republic. Medecine et Maladies Infectieuses. 1989;19(5):322-324.

Hoff NA, Morier DS, Kisalu NK, Johnston SC, Doshi RH, Hensley LE, et al. Varicella coinfection in patients with active monkeypox in the Democratic Republic of the Congo. Ecohealth. 2017;14(3):564-574.

Jezek Z, Arita I, Mutombo M, Dunn C, Nakano JH, Szczeniowski M. Four generations of probable person-to-person transmission of human monkeypox. Am J Epidemiol. 1986;123(6):1004-1012.

Jezek Z, Grab B, Szczeniowski M, Paluku KM, Mutombo M. Clinico-epidemiological features of monkeypox patients with an animal or human source of infection. Bull World Health Organ. 1988;66(4):459-464.

Kalthan E, Dondo-Fongbia JP, Yambele S, Dieu-Creer LR, Zepio R, Pamatika CM. [Twelve cases of monkeypox virus outbreak in Bangassou District (Central African Republic) in December 2015]. Bull Soc Pathol Exot. 2016;109(5):358-363.

Khodakevich L, Widy-Wirski R, Arita I. Monkeypox in the Central African Republic. Bulletin de la Societe de Pathologie Exotique et de ses Filiales. 1985;78(3):311-320.

Learned LA, Reynolds MG, Wassa DW, Li Y, Olson VA, Karem K, et al. Extended interhuman transmission of monkeypox in a hospital community in the Republic of the Congo, 2003. Am J Trop Med Hyg. 2005;73(2):428-434.

McCollum AM, Nakazawa Y, Ndongala GM, Pukuta E, Karhemere S, Lushima RS, et al. Case report: Human monkeypox in the Kivus, a conflict region of the Democratic Republic of the Congo. Am J Trop Med Hyg. 2015;93(4):718-721.

Merouze F, Lesoin JJ. [Monkeypox: second human case observed in Ivory Coast (rural health sector of Daloa]. Med Trop (Mars). 1983;43(2):145-147.

Meyer A, Esposito JJ, Gras F, Kolakowski T, Fatras M, Muller G. [First appearance of monkey pox in human beings in Gabon]. Med Trop (Mars). 1991;51(1):53-57.

Meyer H, Perrichot M, Stemmler M, Emmerich P, Schmitz H, Varaine F, et al. Outbreaks of disease suspected of being due to human monkeypox virus infection in the Democratic Republic of Congo in 2001. J Clin Microbiol. 2002;40(8):2919-2921.

Mwanbal PT, Tshioko KF, Moudi A, Mukinda V, Mwema GN, Messinger D, et al. Human monkeypox in Kasai Oriental, Zaire (1996-1997). Euro Surveill. 1997;2(5):33-35.

Nakoune E, Lampaert E, Ndjapou SG, Janssens C, Zuniga I, Van Herp M, et al. A nosocomial outbreak of human monkeypox in the Central African Republic. Open Forum Infect Dis. 2017;4(4):ofx168.

[No authors listed]. Monkeypox, 1991. Gabon. Wkly Epidemiol Rec. 1992;67(14):101-102.

Nolen LD, Osadebe L, Katomba J, Likofata J, Mukadi D, Monroe B, et al. Extended human-to-human transmission during a monkeypox outbreak in the Democratic Republic of the Congo. Emerg Infect Dis. 2016;22(6):1014-1021.

Reynolds MG, Emerson GL, Pukuta E, Karhemere S, Muyembe JJ, Bikindou A, et al. Detection of human monkeypox in the Republic of the Congo following intensive community education. Am J Trop Med Hyg. 2013;88(5):982-985.

Reynolds MG, Wauquier N, Li Y, Satheshkumar PS, Kanneh LD, Monroe B, et al. Human monkeypox in Sierra Leone after 44-Year absence of reported cases. Emerg Infect Dis. 2019;25(5):1023-1025.

Rimoin AW, Kisalu N, Kebela-Ilunga B, Mukaba T, Wright LL, Formenty P, et al. Endemic human monkeypox, Democratic Republic of Congo, 2001-2004. Emerg Infect Dis. 2007;13(6):934-937.

Rimoin AW, Mulembakani PM, Johnston SC, Lloyd Smith JO, Kisalu NK, Kinkela TL, et al. Major increase in human monkeypox incidence 30 years after smallpox vaccination campaigns cease in the Democratic Republic of Congo. Proc Natl Acad Sci U S A. 2010;107(37):16262-16267.

Tchokoteu PF, Kago I, Tetanye E, Ndoumbe P, Pignon D, Mbede J. [Variola or a severe case of varicella? A case of human variola due to monkeypox virus in a child from the Cameroon]. Ann Soc Belg Med Trop. 1991;71(2):123-128.

World Health Organization. Regional Office for Africa. 2020. Weekly Bulletin on Outbreak and other Emergencies: Week 11: 09 - 15 March 2020. Available from: https://apps.who.int/iris/handle/10665/331451

Ye F, Song J, Zhao L, Zhang Y, Xia L, Zhu L, et al. Molecular evidence of human monkeypox virus infection, Sierra Leone. Emerg Infect Dis. 2019;25(6):1220-1222.

Yinka-Ogunleye A, Aruna O, Dalhat M, Ogoina D, McCollum A, Disu Y, et al. Outbreak of human monkeypox in Nigeria in 2017-18: a clinical and epidemiological report. Lancet Infect Dis. 2019;19(8):872-879.
