# Supplementary material for: The changing epidemiology of human monkeypox—A potential threat? A systematic review
Source: PLoS Negl Trop Dis. 2022 Feb 11;16(2):e0010141. doi: 10.1371/journal.pntd.0010141 (PMC8870502; doi:10.1371/journal.pntd.0010141)
Supplement: S5 Table — (DOCX) [file pntd.0010141.s005.docx]

#### Table S5. Case fatality rate in confirmed, probable, and/or possible monkeypox cases

| Author, year (citation) | Study period | Confirmed, probable or possible cases (n) | Case fatality rate n (%) | Age; gender deceased cases |
| --- | --- | --- | --- | --- |
| *Cameroon* | | | |  |
| Tchokoteu, 1991 (50) | Dec 1989 | 1 | 0 |  |
| *WHO, 2018 (67)* | *April - June 2018* | *1* | *0* |  |
| *WHO, 2020 (65)* | *Sep 2019* | *1* | *0* |  |
| *WHO, 2020 (68)* | *Dec 2019* | *2* | *1 (50)* | *1; NR* |
| *Central African Republic* | | | |  |
| Herve, 1989 (31) | Likely 1980s | 2 | 0 |  |
| Khodakevich, 1985 (34) | Jan 1984 | 6 | 0 |  |
| Berthet, 2011 (29) | June 2010 | 2 | 0 |  |
| Nakouné, 2017 (35); Kalthan 2016 (32) | Dec 2015 - Feb 2016 | 13 | 3 (23.1) | 1, 5 and 26; 3M |
| *WHO, 2017 (60)* | *Feb - April 2017* | *5* | *0* |  |
| *WHO, 2017 (61)* | *April - June 2017* | *2* | *0* |  |
| *WHO, 2019 (59)* | *2 March 2018 - 2 June 2019* | *25* | *3 (12)* |  |
| *Democratic Republic of the Congo* | | | |  |
| Breman, 1980 (5) | 1970-1979 | 38 | 8 (21) | 0-7; 6M, 2F |
| Jezek, 1988 (21) | 1981-1986 | 338 | 33 (9.8) | 0-8; NR |
| Jezek, 1986 (20) | May - July 1983 | 5 | 1 (20) | 1; M |
| Mwanbal, 1997 (13) | Feb 1996 - Feb 1997 | 92 | 3 (3.3) | <3; NR |
| Nolen, 2016 (25) | July - Dec 2013 | 39 | 10 (25.6) |  |
| McCollum, 2015 (23) | 2011-2014 | 3 | likely 0 |  |
| Eltvedt, 2020 (16) | Dec 2016 | 1 | 1 (100) | 4; NR |
| *Gabon* | | | |  |
| Meyer, 1991 (54) | June 1987 | 4 | 2 (50) | 0 and 4; 1F, 1M |
| No authors, 1992 (53) | Jan, May-July 1991 | 9 | 0 |  |
| *Israel* | | | |  |
| Erez, 2019 (57) | Oct 2018 | 1 | 0 |  |
| *Ivory Coast (C*ô*te d’Ivoire)* | | | |  |
| Breman, 1977 (51) | Oct 1972 | 1 | 0 |  |
| Merouze, 1983 (52) | Jan 1981 | 1 | 0 |  |
| *Liberia* | | | |  |
| Foster, 1972 (42) | Sep – Oct 1970 | 4 | 0 |  |
| *WHO, 2018 (73)* | *Nov 2016 – Dec 2017* | *2* | *0* |  |
| *Nigeria* | | | |  |
| Foster, 1972 (42) | April 1971 | 1 | 0 |  |
| Breman, 1980 (5) | April 1971 | 1 | 0 |  |
| Breman, 1980 (5) | Nov 1978 | 1 | 0 |  |
| *Nigeria Centre for Disease Control (74);* Yinka-Ogunleye 2019 (44) | *2017-2019* | *181** | *9 (5.0)* | mean age 27±14 years in seven cases, including one baby |
| *Republic of the Congo* | | | |  |
| Learned, 2005 (46) | April – June 2003 | 11 | 1 (9) | 10; F |
| Reynolds, 2013 (47) | April – Nov 2010 | 2 | 0 |  |
| Doshi, 2019 (15) | Jan – 5 April 2017 | 22 | 3 (13.6) | 4, 14, 40; 1M, 2F |
| *WHO, 2019 (70)* | *March 2019* | *2* | *0* |  |
| *Sierra Leone* | | | |  |
| Foster, 1972 (42) | Dec 1970 | 1 | 0 |  |
| Reynolds, 2019 (48) | March 2014 | 1 | 0 |  |
| Ye, 2019 (49) | March 2017 | 1 | 0 |  |
| *Singapore* | | | |  |
| Yong, 2020 (8) | May 2019 | 1 | 0 |  |
| *South Sudan* | | | |  |
| Formenty, 2010 (58) | Sep – Dec 2005 | 19 | 0 |  |
| *United Kingdom* | | | |  |
| Vaughan, 2018 (55) | Sep 2018 | 2 | 0 |  |
| Vaughan, 2020 (56) | Sep 2018 | 1 | 0 |  |
| *United States* | | | |  |
| *Centers for Disease Control and Prevention (6)* | *2003* | *47* | *0* |  |

#### Note: Citation numbers reflect those that are in the main manuscript text, and those in italics refer to grey

#### literature sources. F = female; M = male.

#### * Two cases diagnosed in the UK were subtracted from the total 183 reported from Nigeria.

**References** (listed in alphabetical order; citation numbers in the Table reflect those that are in the main manuscript text for ease of identification)

Berthet N, Nakouné E, Whist E, Selekon B, Burguière AM, Manuguerra JC, et al. Maculopapular lesions in the Central African Republic. Lancet. 2011;378(9799):1354.

Breman JG, Nakano JH, Coffi E, Godfrey H, Gautun JC. Human poxvirus disease after smallpox eradication. Am J Trop Med Hyg. 1977;26(2):273-281.

Breman JG, Kalisa R, Steniowski MV, Zanotto E, Gromyko AI, Arita I. Human monkeypox, 1970-79. Bull World Health Organ. 1980;58(2):165-182.

Centers for Disease Control and Prevention. Monkeypox. Available from: <https://www.cdc.gov/poxvirus/monkeypox/index.html>

Doshi RH, Guagliardo SAJ, Doty JB, Babeaux AD, Matheny A, Burgado J, et al. Epidemiologic and ecologic investigations of monkeypox, Likouala Department, Republic of the Congo, 2017. Emerg Infect Dis. 2019;25(2):281-289.

Eltvedt AK, Christiansen M, Poulsen A. A case report of monkeypox in a 4-year-old boy from the DR Congo: challenges of diagnosis and management. Case Rep Pediatr. 2020;2020:8572596.

Erez N, Achdout H, Milrot E, Schwartz Y, Wiener-Well Y, Paran N, et al. Diagnosis of imported monkeypox, Israel, 2018. Emerg Infect Dis. 2019;25(5):980-983.

Formenty P, Muntasir MO, Damon I, Chowdhary V, Opoka ML, Monimart C, et al. Human monkeypox outbreak caused by novel virus belonging to Congo Basin clade, Sudan, 2005. Emerg Infect Dis. 2010;16(10):1539-1545.

Foster SO, Brink EW, Hutchins DL, Pifer JM, Lourie B, Moser CR, et al. Human monkeypox. Bull World Health Organ. 1972;46(5):569-576.

Herve VMA, Belec L, Yayah G, Georges AJ. Monkeypox in Central Africa. About two strains isolated Central African Republic. Medecine et Maladies Infectieuses. 1989;19(5):322-324.

Jezek Z, Arita I, Mutombo M, Dunn C, Nakano JH, Szczeniowski M. Four generations of probable person-to-person transmission of human monkeypox. Am J Epidemiol. 1986;123(6):1004-1012.

Jezek Z, Grab B, Szczeniowski M, Paluku KM, Mutombo M. Clinico-epidemiological features of monkeypox patients with an animal or human source of infection. Bull World Health Organ. 1988;66(4):459-464.

Kalthan E, Dondo-Fongbia JP, Yambele S, Dieu-Creer LR, Zepio R, Pamatika CM. [Twelve cases of monkeypox virus outbreak in Bangassou District (Central African Republic) in December 2015]. Bull Soc Pathol Exot. 2016;109(5):358-363.

Khodakevich L, Widy-Wirski R, Arita I. Monkeypox in the Central African Republic. Bulletin de la Societe de Pathologie Exotique et de ses Filiales. 1985;78(3):311-320.

Learned LA, Reynolds MG, Wassa DW, Li Y, Olson VA, Karem K, et al. Extended interhuman transmission of monkeypox in a hospital community in the Republic of the Congo, 2003. Am J Trop Med Hyg. 2005;73(2):428-434.

McCollum AM, Nakazawa Y, Ndongala GM, Pukuta E, Karhemere S, Lushima RS, et al. Case report: Human monkeypox in the Kivus, a conflict region of the Democratic Republic of the Congo. Am J Trop Med Hyg. 2015;93(4):718-721.

Merouze F, Lesoin JJ. [Monkeypox: second human case observed in Ivory Coast (rural health sector of Daloa]. Med Trop (Mars). 1983;43(2):145-147.

Meyer A, Esposito JJ, Gras F, Kolakowski T, Fatras M, Muller G. [First appearance of monkey pox in human beings in Gabon]. Med Trop (Mars). 1991;51(1):53-57.

Mwanbal PT, Tshioko KF, Moudi A, Mukinda V, Mwema GN, Messinger D, et al. Human monkeypox in Kasai Oriental, Zaire (1996-1997). Euro Surveill. 1997;2(5):33-35.

Nakoune E, Lampaert E, Ndjapou SG, Janssens C, Zuniga I, Van Herp M, et al. A nosocomial outbreak of human monkeypox in the Central African Republic. Open Forum Infect Dis. 2017;4(4):ofx168.

Nigeria Centre for Disease Control. Nigeria monkeypox monthly situation report. December 2019.

[No authors listed]. Monkeypox, 1991. Gabon. Wkly Epidemiol Rec. 1992;67(14):101-102.

Nolen LD, Osadebe L, Katomba J, Likofata J, Mukadi D, Monroe B, et al. Extended human-to-human transmission during a monkeypox outbreak in the Democratic Republic of the Congo. Emerg Infect Dis. 2016;22(6):1014-1021.

Reynolds MG, Emerson GL, Pukuta E, Karhemere S, Muyembe JJ, Bikindou A, et al. Detection of human monkeypox in the Republic of the Congo following intensive community education. Am J Trop Med Hyg. 2013;88(5):982-985.

Reynolds MG, Wauquier N, Li Y, Satheshkumar PS, Kanneh LD, Monroe B, et al. Human monkeypox in Sierra Leone after 44-Year absence of reported cases. Emerg Infect Dis. 2019;25(5):1023-1025.

Tchokoteu PF, Kago I, Tetanye E, Ndoumbe P, Pignon D, Mbede J. [Variola or a severe case of varicella? A case of human variola due to monkeypox virus in a child from the Cameroon]. Ann Soc Belg Med Trop. 1991;71(2):123-128.

Vaughan A, Aarons E, Astbury J, Balasegaram S, Beadsworth M, Beck CR, et al. Two cases of monkeypox imported to the United Kingdom, September 2018. Euro Surveill. 2018;23(38).

Vaughan A, Aarons E, Astbury J, Brooks T, Chand M, Flegg P, et al. Human-to-human transmission of monkeypox virus, United Kingdom, October 2018. Emerg Infect Dis. 2020;26(4):782-785.

World Health Organization. Regional Office for Africa, Health Emergencies Programme. 2017. Weekly Bulletin on Outbreaks and other Emergencies: Week 21: 20 – 26 May 2017. Available from: https://apps.who.int/iris/handle/10665/255579

World Health Organization. Regional Office for Africa, Health Emergencies Programme. 2017. Weekly Bulletin on Outbreak and other Emergencies: Week 31: 29 July – 04 August 2017. Available from: https://apps.who.int/iris/handle/10665/258688

World Health Organization. Regional Office for Africa, Health Emergencies Programme. 2018. Weekly Bulletin on Outbreaks and other Emergencies: Week 1: 30 December 2017 - 5 January 2018. Available from: https://apps.who.int/iris/handle/10665/259809

World Health Organization. Regional Office for Africa. 2018. Weekly Bulletin on Outbreak and other Emergencies: Week 31: 28 July - 3 August 2018. Available from: https://apps.who.int/iris/handle/10665/273631

World Health Organization. Regional Office for Africa. 2019. Weekly Bulletin on Outbreak and other Emergencies: Week 21: 20 - 26 May 2019. Available from: https://apps.who.int/iris/handle/10665/324950

World Health Organization. Regional Office for Africa. 2019. Weekly Bulletin on Outbreak and other Emergencies: Week 31: 29 July - 04 August 2019. Available from: https://apps.who.int/iris/handle/10665/326159

World Health Organization. Regional Office for Africa. 2020. Weekly Bulletin on Outbreak and other Emergencies: Week 01: 30 December 2019 - 05 January 2020. Available from: https://apps.who.int/iris/handle/10665/330353

World Health Organization. Regional Office for Africa. 2020. Weekly Bulletin on Outbreak and other Emergencies: Week 11: 09 - 15 March 2020. Available from: https://apps.who.int/iris/handle/10665/331451

Ye F, Song J, Zhao L, Zhang Y, Xia L, Zhu L, et al. Molecular evidence of human monkeypox virus infection, Sierra Leone. Emerg Infect Dis. 2019;25(6):1220-1222.

Yinka-Ogunleye A, Aruna O, Dalhat M, Ogoina D, McCollum A, Disu Y, et al. Outbreak of human monkeypox in Nigeria in 2017-18: a clinical and epidemiological report. Lancet Infect Dis. 2019;19(8):872-879.

Yong SEF, Ng OT, Ho ZJM, Mak TM, Marimuthu K, Vasoo S, et al. Imported monkeypox, Singapore. Emerg Infect Dis. 2020;26(8):1826-1830.
