# Supplementary material for: The changing epidemiology of human monkeypox—A potential threat? A systematic review
Source: PLoS Negl Trop Dis. 2022 Feb 11;16(2):e0010141. doi: 10.1371/journal.pntd.0010141 (PMC8870502; doi:10.1371/journal.pntd.0010141)
Supplement: S6 Table — (DOCX) [file pntd.0010141.s006.docx]

#### Table S6. Transmission of monkeypox

| Author, year (citation) | Study period | Number of confirmed, probable and/or possible cases | Mode of transmission | Transmission details |
| --- | --- | --- | --- | --- |
| *Cameroon* | | | | |
| Breman, 1980 (5) | 1979 | 1 | Unknown | Contact with a dead squirrel about two weeks before the illness |
| *Central African Republic* | | | | |
| Khodakevich, 1985 (34) | Jan 1984 | 6 | Animal-to-human | A sick animal was eaten. |
| Berthet, 2011 (29) | June 2010 | 2 | Likely animal-to-human | The lesions of both cases developed after hunting and eating a wild rodent |
| Nakouné, 2017 (35) | Dec 2015 – Jan 2016 | 10 | Both | The index case fell sick after killing and cutting up a rodent. Although the authors cannot be 100% certain, the nurses, other members of the family, and different ferryboat drivers were probably contaminated by either the index case or case No. 2 (brother of index case who got sick five days after index case) and not between themselves. |
| Kalthan, 2018 (33) | Aug – Oct 2016 | 3 plus 23 suspected cases | Both | The index case was a hunter and farmer. He had consumed meat that came from the Xerus erythropus species of squirrels, found dead in the forest. All but four patients were secondary cases. |
| Besombes, 2019 (30) | Sep – Oct 2018 | 6 | Both | The index case-patient reported butchering 3 small mammals. The infection of the other 5 family members is considered human-to human transmission. |
| *Democratic Republic of the Congo* | | | | |
| Breman, 1980 (5) | 1970-1979 | 38 | Both | Person-to-person spread might have occurred in three cases. In four instances, presumed co-primary cases occurred in the same family. |
| Jezek, 1988 (21) | 1981- 1986 | 338 | Both | Animal source: suspected in 245 cases (72.5%); human source in 93 cases (27.5%). |
| Jezek, 1986 (20) | May – July 1983 | 5 | Both | Case 1 ate monkey (rest of the family as well). Circumstances suggest that case 1 infected case 2 and case 2 infected case 3. They belong to one family. Case 4 ate Gambian pouched rat (rest of the family as well). Possibly case 4 was however infected by case 3 by accident in the hospital. Case 5 was likely infected by case 4 (family) |
| Aplogan, 1997 (14) | Feb 1996 – Feb 1997 | 419 | Both | Secondary cases: 147 (35%) reported having travelled outside their home village during the 3 weeks preceding disease onset. Of the secondary cases, 53% reported having had antecedent contact with another case-patient in the neighbourhood, 48% in the housing compound, and 42% in an individual household. Primary cases with no apparent association with the clusters in the Akungula/Ekanga occurred in 49 of the 78 affected villages. |
| Meyer, 2002 (24) | Feb - Aug 2001 | 7 | Animal-to-human, and not determined | Outbreak 7: might have been a monkey found dead in the forest that was handled and eaten by the family |
| Nolen, 2016 (25) | Jul – Dec 2013 | 39 |  | Nine families showed >1 transmission event, and >6 transmission events occurred within this health zone. |
| McCollum, 2015 (23) | 2011 – 2014 | 3 | Likely all animal-to-human | Case 2: had handled monkeys killed by local hunters, saved monkey meat for his voyage, and then ate this meat over the course of his travel. Case 1 also noted contact with bushmeat before illness onset. |
| *Gabon* | | | | |
| Meyer, 1991 (54) | June 1987 | 4 | Unknown | Epidemiological study did not reveal any human transmission evidence, nor the source of infection. |
| No authors, 1992 (53) | Jan, May -July 1991 | 9 | Unknown | Cats, dogs, rats, and mice were found in the homes. In one case a small monkey had been taken into the home a few days before onset of illness. It was not reported whether these could have transmitted MPX. |
| *Israel* | | | | |
| Erez, 2019 (57) | Oct 2018 | 1 | Likely animal | Case disposed 2 rodent carcasses at his residence. |
| *Ivory Coast (C*ô*te d’Ivoire)* | | | | |
| Merouze, 1983 (52) | Jan 1981 | 1 | Unknown | Not possible to prove either animal-human or interhuman contagion |
| *Liberia* | | | | |
| Foster, 1972 (42) | Sep 1970 | 1 | Unknown | Playmates with other two cases from Boudua. All three were observed to play with internal organs removed from recently killed monkeys. No evidence of definite monkey contact could be established. No evidence of definite exposure to sick animals, wild or domestic. No exposure to a sick person, either resident or visitor, could be recalled by any of the residents |
|  | Sep 1970 | 1 | Unknown | Playmates with other two cases from Boudua. All three were observed to play with internal organs removed from recently killed monkeys. No evidence of definite monkey contact could be established. No evidence of definite exposure to sick animals, wild or domestic. No exposure to a sick person, either resident or visitor, could be recalled by any of the residents |
|  | Sep 1970 | 1 | Unknown | Playmates with other two cases from Boudua. All three were observed to play with internal organs removed from recently killed monkeys. No evidence of definite monkey contact could be established. No evidence of definite exposure to sick animals, wild or domestic. No exposure to a sick person, either resident or visitor, could be recalled by any of the residents |
|  | Oct 1970 | 1 | Unknown | Occasionally consumed freshly killed monkeys for food. No evidence of definite monkey contact could be established. No evidence of definite exposure to sick animals, wild or domestic. |
| *Nigeria* | | | | |
| Foster, 1972 (42) | April 1971 | 1 | Unknown | No evidence of definite exposure to sick animals, wild or domestic, could be obtained. Monkey exposure was highly unlikely. The case had a limited exposure to domestic animals, including dogs, cats, poultry, sheep, goats, and pigs. Residential or farm contact with household rodents and bats was also possible. |
| Breman, 1980 (5) | April 1971 | 1 | Human-to-human | Secondary transmission presumed, mother of other case from Nigeria, April 1971. |
| Yinka-Ogunleye, 2019 (44) | Sep 2017 – Sep 2018 | 122 | Both | Of the 122 cases 36 (30%) had an epidemiological link with people with similar lesions before the onset of monkeypox. Of these 36 people, 12 (33%) were epidemiologically linked with a confirmed case. Seven (58%) of these 12 people shared a household or had intimate contact with a confirmed case, four (33%) were inmates in the same prison as a confirmed case, and one was a health worker who treated a confirmed case. Among all confirmed cases, 10 patients reported contact with animals (two with monkeys, two with rodents, two with unspecified wild animal [consumed as meat—ie, bush meat], and four with domestic animals). No one reported contact with sick or dead animals. |
| *Republic of the Congo* | | | | |
| Learned, 2005 (46) | April – June 2003 | 11 | Most human-to-human | Case 0 was unavailable for an interview; therefore, the nature of his potential exposures to monkeypox virus is not known. Monkeypox was likely imported with the visit of case 0. Case 1 did own a pet monkey. On visual inspection, the animal appeared to be in good health, and the family could not recall its having been ill within the previous four months. No other potential wild animal exposures were reported for this child, or for the other patients described. The approximate case interval between cases 0 and 1 is consistent with previous descriptions of person-to-person transmission of monkeypox. All subsequent confirmed, probable, and suspect cases in the outbreak had epidemiologic linkage to the Government Hospital in Impfondo, suggesting all further infections were human-to-human. |
| Reynolds, 2013 (47) | April – Nov 2010 | 2 | Unknown | Insufficient evidence. The uncle of the 16-year-old girl and his two sons were reported to have had similar illnesses during the previous month; the uncle died and the two boys had recovered by the time the girl fell ill |
| Doshi, 2019 (15) | Jan – April 2017 | 22 | Both and unknown | Three separate clusters, each in one district: Eyelle: exposure of two cases unknown, 1 likely human-to-human. Dongou: First case was a hunter. For one case exposure was unknown. Six others were hunters but also had contact with cases. The final seven were contacts/family clusters. Impfondo: first case prepared bush meat. Other three were family members. |
| *Sierra Leone* | | | | |
| Foster, 1972 (42) | Dec 1970 | 1 | Unknown | No evidence of definite exposure to sick animals, wild or domestic. No evidence of definite monkey contact could be established in the 3 weeks prior to the onset of rash disease. |
| Reynolds, 2019 (48) | March 2014 | 1 | Unknown | The child’s mother denied that he had had any contact with persons exhibiting a monkeypox-like illness in the 2 weeks before onset of his illness. The mother also denied that the child had had any history of contact with animals. However, both the mother and father of the boy stated that they regularly prepare and consume meat from wild animals. The mother and father also confirmed that small rodents were sometimes present in the family house. |
| Ye, 2019 (49) | March 2017 | 1 | Animal | The patients had been hunting and eating squirrels ≈10 days before becoming ill, and traveling to Pelewahun gee bu in Bo district 3 days before symptoms began. |
| *Singapore* | | | | |
| Yong, 2020 (8) | May 2019 | 1 | Likely animal | Ingestion of barbecued bushmeat that might have been contaminated. Patient did not handle raw meat and had no exposure to wild animals or their products, had no contact with rodents or with persons with pox-like illnesses. |
| *South Sudan* | | | | |
| Formenty, 2010 (58) | Sep – Dec 2005 | 19 | Human-to-human and unknown | Person-to-person transmission was documented in 3 chains associated with the activities of a traditional healer and tooth extractor. Fourteen case-patients reported contact with a suspected monkeypox case-patient before onset of symptoms; 1 case-patient was probably exposed to infected material during his hospitalization at the MSF-F hospital; and 6 case-patients did not report any known likely mode of infection. Among those 6, 3 reported that the rash began appearing around a pre-existing wound, which could have been from the bite of an infected animal. None reported contact with wild animals. |
| *United Kingdom* | | | | |
| Vaughan, 2018 (55) | Sep 2018 | 2 | Unknown and likely human-to-human | Case 2: contact with an individual with a monkeypox-like rash at a large family event and consumption of bush meat during his visit to a rural area of Nigeria. |
| Vaughan, 2020 (56) | Sep 2018 | 1 | Human-to-human | The only exposure risk identified during assessment was the changing of potentially contaminated bedding, when case 2 had multiple skin lesions, but before a diagnosis of monkeypox had been considered. |
| *United States* | | | | |
| Reynolds, 2007 (41); *CDC (6*) | 2003 | 47 | Animal-to-human | All reported exposure to ill or infected prairie dogs |

#### Note: Citation numbers reflect those that are in the main manuscript text, and those in italics refer to grey literature sources. CDC= United States Centers for Disease Control and Prevention; MPX = monkeypox.

**References** (listed in alphabetical order; citation numbers in the Table reflect those that are in the main manuscript text for ease of identification)

Aplogan A, Mangindula V, Muamba PT, Mwema GN, Okito L, Pebody RG, et al. Human monkeypox -- Kasai Oriental, Democratic Republic of Congo, February 1996-October 1997. MMWR Morb Mortal Wkly Rep. 1997;46(49):1168-1171.

Berthet N, Nakouné E, Whist E, Selekon B, Burguière AM, Manuguerra JC, et al. Maculopapular lesions in the Central African Republic. Lancet. 2011;378(9799):1354.

Besombes C, Gonofio E, Konamna X, Selekon B, Grant R, Gessain A, et al. Intrafamily transmission of monkeypox virus, Central African Republic, 2018. Emerg Infect Dis. 2019;25(8):1602-1604.

Breman JG, Kalisa R, Steniowski MV, Zanotto E, Gromyko AI, Arita I. Human monkeypox, 1970-79. Bull World Health Organ. 1980;58(2):165-182.

Centers for Disease Control and Prevention. Monkeypox. Available from: <https://www.cdc.gov/poxvirus/monkeypox/index.html>

Doshi RH, Guagliardo SAJ, Doty JB, Babeaux AD, Matheny A, Burgado J, et al. Epidemiologic and ecologic investigations of monkeypox, Likouala Department, Republic of the Congo, 2017. Emerg Infect Dis. 2019;25(2):281-289.

Erez N, Achdout H, Milrot E, Schwartz Y, Wiener-Well Y, Paran N, et al. Diagnosis of imported monkeypox, Israel, 2018. Emerg Infect Dis. 2019;25(5):980-983.

Formenty P, Muntasir MO, Damon I, Chowdhary V, Opoka ML, Monimart C, et al. Human monkeypox outbreak caused by novel virus belonging to Congo Basin clade, Sudan, 2005. Emerg Infect Dis. 2010;16(10):1539-1545.

Foster SO, Brink EW, Hutchins DL, Pifer JM, Lourie B, Moser CR, et al. Human monkeypox. Bull World Health Organ. 1972;46(5):569-576.

Jezek Z, Arita I, Mutombo M, Dunn C, Nakano JH, Szczeniowski M. Four generations of probable person-to-person transmission of human monkeypox. Am J Epidemiol. 1986;123(6):1004-1012.

Jezek Z, Grab B, Szczeniowski M, Paluku KM, Mutombo M. Clinico-epidemiological features of monkeypox patients with an animal or human source of infection. Bull World Health Organ. 1988;66(4):459-464.

Kalthan E, Tenguere J, Ndjapou SG, Koyazengbe TA, Mbomba J, Marada RM, et al. Investigation of an outbreak of monkeypox in an area occupied by armed groups, Central African Republic. Med Mal Infect. 2018;48(4):263-268.

Khodakevich L, Widy-Wirski R, Arita I. Monkeypox in the Central African Republic. Bulletin de la Societe de Pathologie Exotique et de ses Filiales. 1985;78(3):311-320.

Learned LA, Reynolds MG, Wassa DW, Li Y, Olson VA, Karem K, et al. Extended interhuman transmission of monkeypox in a hospital community in the Republic of the Congo, 2003. Am J Trop Med Hyg. 2005;73(2):428-434.

McCollum AM, Nakazawa Y, Ndongala GM, Pukuta E, Karhemere S, Lushima RS, et al. Case report: Human monkeypox in the Kivus, a conflict region of the Democratic Republic of the Congo. Am J Trop Med Hyg. 2015;93(4):718-721.

Merouze F, Lesoin JJ. [Monkeypox: second human case observed in Ivory Coast (rural health sector of Daloa]. Med Trop (Mars). 1983;43(2):145-147.

Meyer A, Esposito JJ, Gras F, Kolakowski T, Fatras M, Muller G. [First appearance of monkey pox in human beings in Gabon]. Med Trop (Mars). 1991;51(1):53-57.

Meyer H, Perrichot M, Stemmler M, Emmerich P, Schmitz H, Varaine F, et al. Outbreaks of disease suspected of being due to human monkeypox virus infection in the Democratic Republic of Congo in 2001. J Clin Microbiol. 2002;40(8):2919-2921.

Nakoune E, Lampaert E, Ndjapou SG, Janssens C, Zuniga I, Van Herp M, et al. A nosocomial outbreak of human monkeypox in the Central African Republic. Open Forum Infect Dis. 2017;4(4):ofx168.

[No authors listed]. Monkeypox, 1991. Gabon. Wkly Epidemiol Rec. 1992;67(14):101-102.

Nolen LD, Osadebe L, Katomba J, Likofata J, Mukadi D, Monroe B, et al. Extended human-to-human transmission during a monkeypox outbreak in the Democratic Republic of the Congo. Emerg Infect Dis. 2016;22(6):1014-1021.

Reynolds MG, Davidson WB, Curns AT, Conover CS, Huhn G, Davis JP, et al. Spectrum of infection and risk factors for human monkeypox, United States, 2003. Emerg Infect Dis. 2007;13(9):1332-1339.

Reynolds MG, Emerson GL, Pukuta E, Karhemere S, Muyembe JJ, Bikindou A, et al. Detection of human monkeypox in the Republic of the Congo following intensive community education. Am J Trop Med Hyg. 2013;88(5):982-985.

Reynolds MG, Wauquier N, Li Y, Satheshkumar PS, Kanneh LD, Monroe B, et al. Human Monkeypox in Sierra Leone after 44-Year Absence of Reported Cases. Emerg Infect Dis. 2019;25(5):1023-1025.

Vaughan A, Aarons E, Astbury J, Balasegaram S, Beadsworth M, Beck CR, et al. Two cases of monkeypox imported to the United Kingdom, September 2018. Euro Surveill. 2018;23(38).

Vaughan A, Aarons E, Astbury J, Brooks T, Chand M, Flegg P, et al. Human-to-human transmission of monkeypox virus, United Kingdom, October 2018. Emerg Infect Dis. 2020;26(4):782-785.

Ye F, Song J, Zhao L, Zhang Y, Xia L, Zhu L, et al. Molecular evidence of human monkeypox virus infection, Sierra Leone. Emerg Infect Dis. 2019;25(6):1220-1222.

Yinka-Ogunleye A, Aruna O, Dalhat M, Ogoina D, McCollum A, Disu Y, et al. Outbreak of human monkeypox in Nigeria in 2017-18: a clinical and epidemiological report. Lancet Infect Dis. 2019;19(8):872-879.

Yong SEF, Ng OT, Ho ZJM, Mak TM, Marimuthu K, Vasoo S, et al. Imported monkeypox, Singapore. Emerg Infect Dis. 2020;26(8):1826-1830.
