# Supplementary material for: The changing epidemiology of human monkeypox—A potential threat? A systematic review
Source: PLoS Negl Trop Dis. 2022 Feb 11;16(2):e0010141. doi: 10.1371/journal.pntd.0010141 (PMC8870502; doi:10.1371/journal.pntd.0010141)
Supplement: Attachment — Submitted filename: response letter_v1_8Nov2021.docx [file pntd.0010141.s007.docx]

Drs. Gregory Gromowski and A. Desiree LaBeaud
Editors, PLOS Neglected Tropical Diseases

November xx, 2021

Dear Drs. Gromowski and LaBeaud:

We thank the editors and reviewers for their review of our manuscript entitled ‘The changing epidemiology of human monkeypox – a potential threat? A systematic review’ (PNTD-D-21-00919) for consideration of publication in *PLOS Neglected Tropical Diseases.*

We appreciate the thoughtful comments and feedback, and the opportunity to submit a revised version of the manuscript to *PLOS Neglected Tropical Diseases*. We believe we have addressed the concerns of the reviewers and made the appropriate changes to the manuscript. Our detailed response to each reviewer point raised is found below.

*Methods*

*Reviewer #1: It was a well designed systematic review of the literature on monkeypox that was registered with PROSPERO*

*Reviewer #2: The manuscript presents a systematic review of monkeypox epidemiology with a focus on changes in the evolution since the first cases in the 1970s through the time of research. The review followed the PRISMA guideline and was prospectively registered with PROSTERO. The methods are well-explained. The study design, defined population (Africa and Ex-Africa) and analysis are appropriate for the study objective. Competing interest by the funder and initiator of the study, Bavarian Nordic (manufacturers of monkeypox vaccines) and the authors are declared.*

We thank the reviewers for the positive feedback.

*Results*

*Reviewer #1: The analysis was thorough, informative and well presented. The information presented is timely.*

We thank the reviewer for the positive feedback

*Reviewer #2: The analysis is descriptive and matches the analysis plan. However, there are some missing components that are critical.*

*Given the focus on describing the epidemiology of monkeypox, one gap was an absence of an analysis of genomics of the virus. An important recommendation for the authors to consider is an appraisal of the genetic evolution of the virus. Some studies to consider include: https://eur02.safelinks.protection.outlook.com/?url=https%3A%2F%2Fdoi.org%2F10.1093%2Finfdis%2Fjiaa559&amp;data=04%7C01%7Cbeho%40bavarian-nordic.com%7C45505ce6d7c04be44d9f08d9932a3668%7C8122ecda199447d3aed5367de0a7cb9c%7C0%7C0%7C637702631789454264%7CUnknown%7CTWFpbGZsb3d8eyJWIjoiMC4wLjAwMDAiLCJQIjoiV2luMzIiLCJBTiI6Ik1haWwiLCJXVCI6Mn0%3D%7C1000&amp;sdata=Nz1sObsxfVCTeb%2FTsPkoXCKoY4xgrrufAyWv%2FHUrP2s%3D&amp;reserved=0 ; https://eur02.safelinks.protection.outlook.com/?url=https%3A%2F%2Fdoi.org%2F10.3201%2Feid2002.130118&amp;data=04%7C01%7Cbeho%40bavarian-nordic.com%7C45505ce6d7c04be44d9f08d9932a3668%7C8122ecda199447d3aed5367de0a7cb9c%7C0%7C0%7C637702631789454264%7CUnknown%7CTWFpbGZsb3d8eyJWIjoiMC4wLjAwMDAiLCJQIjoiV2luMzIiLCJBTiI6Ik1haWwiLCJXVCI6Mn0%3D%7C1000&amp;sdata=bDIW4fXn1O%2Bv8Rm1BLbUqjjGhu%2FfmdCS0lH0GLTxAa0%3D&amp;reserved=0, https://eur02.safelinks.protection.outlook.com/?url=https%3A%2F%2Fdoi.org%2F10.1016%2FS1473-3099&amp;data=04%7C01%7Cbeho%40bavarian-nordic.com%7C45505ce6d7c04be44d9f08d9932a3668%7C8122ecda199447d3aed5367de0a7cb9c%7C0%7C0%7C637702631789454264%7CUnknown%7CTWFpbGZsb3d8eyJWIjoiMC4wLjAwMDAiLCJQIjoiV2luMzIiLCJBTiI6Ik1haWwiLCJXVCI6Mn0%3D%7C1000&amp;sdata=fRIYVVT4arbE7Dx39ndbWdDEcckpbKHZvvORSgYTZc8%3D&amp;reserved=0(18)30043-4*

We thank the reviewer for this thoughtful comment and agree that a link between the genetic evolution of the monkeypox virus and a resurgence in monkeypox cases is an interesting hypothesis. In response, we have added information about this potential link to the Discussion section of the manuscript (pages 26-27, lines 488-493) and are citing one of the publications (Kugelman 2014, reference 85) suggested by the reviewer. In addition, we are now mentioning in the “Strengths and Limitations” section of the Discussion that a review of literature on the genetic evolution of the monkeypox virus was not within the scope of our work (page 29, lines 537-539).

*Conclusions*

*Reviewer #1: Conclusions were well supported by the results cited. Resurgence of monkeypox is an important public health issue.*

*Reviewer #2: The conclusion emphasised the available information and the need for improved surveillance to better understand the epidemiology of the disease*

We thank the reviewers for the positive feedback.

*Editorial and Data Presentation Modifications*

*Reviewer #1: One minor issue concerns the authors statement on lines 483-484 that unvaccinated individuals accounted for approximately 80-96% of monkeypox cases. The authors should explain and document in more detail how this percentage range was determined.*

We thank the reviewer for this comment. In response, we are stating now in the Results section (page 21, lines 354-355) that “the proportion of monkeypox cases with a history of prior smallpox vaccination ranged from 4 – 21%, illustrating that the majority of cases (approximately 80 – 96%) occurred in unvaccinated individuals.”

*Reviewer #2: Figure 2,3,4,5,6 would be better presented in a table for better readability*

We thank the reviewer for this comment. We feel that a visual representation of the number of monkeypox cases using maps provides an advantage to the reader to gain a better picture of the proximity of monkeypox between and among the countries in Africa as well as the spread of the disease outside of Africa. A table, as you have suggested, providing a good snapshot of cases per country over time is shown below. We have added this table to the manuscript as supporting information (S1 Table).

|  | 1970-1979 | 1980-1989 | 1990-1999 | 2000-2009 | 2010-2019 |
| --- | --- | --- | --- | --- | --- |
|  | Number of Confirmed Cases | | | | |
| Africa |  |  |  |  |  |
| DRC | 38 | 343 | 511 | 10,027* | 18,788* |
| Nigeria | 3 | — | — | — | 181 |
| Liberia | 4 | — | — | — | 6 |
| Cameroon | 1 | 1 | — | — | 3 |
| Côte d’Ivoire | 1 | 1 | — | — | — |
| Sierra Leone | 1 | — | — | — | 2 |
| Gabon | — | 4 | 9 | — | — |
| Central African Republic | — | 8 | — | — | 61 |
| Congo | — | — | — | 73 | 24 |
| South Sudan | — | — | — | 19 | — |
| Other Continents |  |  |  |  |  |
| United States | — | — | — | 47 | — |
| United Kingdom | — | — | — | — | 4 |
| Israel | — | — | — | — | 1 |
| Singapore | — | — | — | — | 1 |

* Number reflects suspected cases, since as of the year 2000, the number of suspected cases was primarily reported by the DRC.

*Line 318 - article should be articles*

Since only one article is cited, we have kept the word “article” singular.

*Line 419-421 - The statement " Conflicting findings of significance, however, were reported regarding direct exposure or touching the animal or having received a bite or scratch sufficient to break the skin" is not clear. Please, state the conflict being referred to. Though this is mentioned in the discussion, it needs to be stated in the results.*

We thank the reviewer for this comment. While some of the reported results appear contradictory, this is not the main message of the cited publication. We have deleted this sentence in the Discussion section, and the paragraph ending (page 29, lines 539-543) now reads: “Lastly, data on risk factors for acquiring monkeypox are rather scarce, and some incongruous results were found. In one study, for example, eating duiker and preparing wild animal for food were identified as protective factors [26], which appears in contrast to factors identified in animal-to-human transmission cases [15, 29]. Thus, more formal studies of risk factors are warranted.” We have also reworded the sentence in the Results (page 23, lines 420-422).

*References 38 and 39 are same - please, edit.*

References 38 and 39 differ slightly. Please see the highlighted areas below.

38.	Update: multistate outbreak of monkeypox--Illinois, Indiana, Kansas, Missouri, Ohio, and Wisconsin, 2003. MMWR Morbidity and mortality weekly report. 2003;52(26):616-8. Epub 2003/07/05. PubMed PMID: 12844080.

39.	Update: multistate outbreak of monkeypox--Illinois, Indiana, Kansas, Missouri, Ohio, and Wisconsin, 2003. MMWR Morbidity and mortality weekly report. 2003;52(24):561-4. Epub 2003/06/21. PubMed PMID: 12816106.

*Summary and General Comments

Reviewer #1: This was a well-crafted, comprehensive review of the epidemiology of monkeypox which constitutes a valuable contribution to the field.*

We thank the reviewer for the positive feedback.

*Reviewer #2: This is a relevant study which summarises available evidence of monkeypox epidemiology at a time of increasing awareness of ongoing transmission in Africa and exportation of cases with need for development of control strategies. Though a similar review was published in 2018 (cited), this study marginally more informational by including newly published works. Studies with phylogenetic analysis are excluded. Phylogenetic analysis is important to describe the evolution of monkeypox in the past 4-5 decades.*

We thank the reviewer for the positive feedback and thoughtful comment. As noted above, we agree that a link between the genetic evolution of the monkeypox virus and a resurgence in monkeypox cases is an interesting hypothesis. In response, we have added information about this potential link to the Discussion section of the manuscript (pages 26-27, lines 488-493) and are citing one of the publications (Kugelman 2014, reference 85) suggested by the reviewer. In addition, we are now mentioning in the “Strengths and Limitations” section of the Discussion that a review of literature on the genetic evolution of the monkeypox virus was not within the scope of our work (page 29, lines 537-539).

We again thank the reviewers for their useful comments, which helped improve the manuscript. We hope you agree that the manuscript is now acceptable for publication in *PLOS Neglected Tropical Diseases*. We look forward to your response.

Yours sincerely,

Bernard Hoet

(in the name of the authors)
